# Primary Emotional Systems and Personality: An Evolutionary Perspective

**DOI:** 10.3389/fpsyg.2017.00464

**Published:** 2017-04-11

**Authors:** Christian Montag, Jaak Panksepp

**Affiliations:** ^1^Key Laboratory for NeuroInformation/Center for Information in Medicine, School of Life Science and Technology, University of Electronic Science and Technology of ChinaChengdu, China; ^2^Institute of Psychology and Education, Ulm UniversityUlm, Germany; ^3^Department of Integrative Physiology and Neuroscience, College of Veterinary Medicine, Washington State University, PullmanWA, USA

**Keywords:** emotions, personality, Panksepp, Affective Neuroscience, primary emotional systems

## Abstract

The present article highlights important concepts of personality including stability issues from the perspective of situational demands and stability over the life-course. Following this more introductory section, we argue why individual differences in primary emotional systems may represent the phylogenetically oldest parts of human personality. Our argumentation leads to the need to increasingly consider individual differences in the raw affects/emotions of people to understand human personality in a bottom–up fashion, which can be coordinated with top–down perspectives. In support of this idea, we also review existing evidence linking individual differences in primal emotions as assessed with the Affective Neuroscience Personality Scales and the widely accepted Big Five Model of Personality. In this context, we provide additional evidence on the link between primal emotions and personality in German and Chinese sample populations. In short, this article addresses evolutionary perspectives in the evaluation of human personality, highlighting some of the ancestral emotional urges that probably still control variations in the construction of human personality structures. Moreover, we address how individual differences in primary emotional systems can illuminate linkages to major human psychopathologies and the potential advantages and disadvantages of carrying a certain personality trait within certain cultural/environmental niches.

## Defining Personality

Many definitions of personality exist and we will not aspire to present them all in this paper. Instead, we will seek to construct a synthetic synopsis of various major definitions: Personality describes stable individual differences in cognitive, emotional and motivational aspects of mental states that result in stable behavioral action (especially emotional) tendencies of humans and other animals, but thoughts remain almost impossible to study in animals (in contrast, the study of emotional behaviors and feelings is straightforward (Panksepp, 1998, 2015a,b). Most theorists, in the context of cognition, refer to personality factors being manifested in *stable* thinking patterns.

The stability issue of personality has been discussed in two areas: (i) time stability and (ii) stability over situations. Many longitudinal studies demonstrated that personality is rather stable, in particular starting from early adulthood, when upper brain regions have matured— e.g., when the prefrontal cortex is able to hold a tight grip on emotions arising from phylogenetically old brain areas (see a short overview by Costa and McCrae, 1994; McCrae and Costa, 1994). A review by Edmonds et al. (2008, p. 410) also emphasized the conclusion that personality is stable over time. For instance, they write “in the absence of an energetic attempt to change your personality… in 10 years you will most likely see the same person that you see now.”

Of course, the situational-stability perspective on human personality is somewhat different than the time-stability topic. For many decades, the *personality/consistency paradox* gave researchers headaches (Mischel, 2004), because persons do not behave as stable across different situations as many scientists originally proposed. The *personality paradox* has been partly solved by Mischel and Shoda (1995) and Mischel (2004) using so called *if-then* functions. By using such *if-then* functions it becomes better explainable why someone does not behave with consistent personality patterns in any imaginable situation. These *if-then* functions take into account that stability of a person is dependent on situational characteristics. This can be illustrated by a person showing on the one hand stable conscientious behavior at the job, but on the other hand that may not translate to the home environment. So *if* a person is on the job, *then* he/she acts conscientious. *If* a person is at home, *then* he/she acts not conscientious (or comparably less than on the job). Ultimately this means that same scores in a self-report measure for personality may relate to different environmental mind sets, which may cause confusion when a person is filling in a questionnaire (e.g., I am thinking of my conscientious behavior when doing the dishes). See for more and additional detailed elaborations on the above mentioned examples in the latent state-trait-theory, which “starts from the premise that human cognition, emotion, and behavior depend systematically on characteristics of the person (traits), characteristics of the situation, and the interaction between person and situation” (Steyer et al., 1999; p. 391).

So far, we have noted that personality refers to stable individual differences in emotionality, motivation and cognition resulting in behavioral action patterns. Now, we would like to focus on the emotional parts of personality, because we are convinced that emotional parts are (i) the evolutionary oldest parts of human personality and (ii) drive human personality and behavior in a bottom–up fashion. This will be explained by a short review of what we have already published on this topic/mentioned elsewhere (see Davis and Panksepp, 2011; Montag, 2014; Montag and Reuter, 2014) which will then be deepened and enlarged by new data giving additional insights into the role of emotions into personality. Before we will follow that path in our article, we would first shortly highlight why the study of human personality is of tremendous importance.

## The Importance of Emotions in the Study of Personality

Until today many researchers are joining this fascinating research endeavor trying to understand why humans differ from each other and even in trying to find answers to questions such as “Why am I the type of person, I am?”. So one would think that human curiosity would be enough to answer why humans and many researchers are interested in this research topic. Of course there are many other issues to consider.

Besides nourishing human curiosity there are many hard facts showing that a better understanding of human personality is of great importance for promoting human welfare. Potentially the most important reason for the study of human personality can be found in the areas of psychiatry and well-being. It has been shown in many studies that negative emotionality, especially with respect to the personality dimension of Neuroticism represents a risk factor for instigating and promoting affective disorders (Lahey, 2009). Thus we can anticipate that the affective disentangling of the biological basis of this important personality trait will eventually result in a better understanding of the molecular foundations of Neuroticism as well as many other personality disorders (Karterud et al., 2016). This might facilitate development of new psychopharmaceuticals to better treat affective disorders (e.g., Panksepp, 2015b, 2016). The study of the “healthy neurotic person” may also promote our understanding of anxiety disorders, because one can assume that dysbalances of neurotransmitters/neuropeptides seen in many affective disorders, will be absent or only present to a lesser degree in healthy-normal variations of human personality. Of course, simply focussing on the study of individual differences in negative emotionality is a focus that is far too narrow to understand the emotional breadth of personality. Thus, we fear that the past has seen a too strong focus on the study of individual differences in negative emotionality; indeed, a better understanding of the neuro-biological/psychological basis of positive emotionality will perhaps be of even greater importance to make progress in the development of better treatments for disorders characterized by negative affect, especially depression.

Aside from some progresses in psychiatry due to personality research, many other important life outcomes can be successfully linked to human personality emphasizing the importance of this research endeavor. To name a few: longevity (Jackson et al., 2015), health behavior (Bogg and Roberts, 2004, 2013) financial decision making (Lauriola and Levin, 2001; Bibby and Ferguson, 2011) and job performance (Barrick and Mount, 1991).

## Primal Emotions As Phylogenetically Oldest Part of Human Personality?

Considering the architecture of the human brain, it becomes obvious that individual differences in emotionality could represent the evolutionary oldest part of human personality (a fact that synergizes with the classical division of personality into *choleric, melancholic, phlegmatic*, and *sanguine* types; see Stelmack and Stalikas, 1991). Following the ideas of *MacLean’s Triune Brain Concept*, the human brain can be divided into at least three major evolutionary passages – at minimum – layers designated as the reptilian (deep subcortical), old-mammalian (limbic) and neo-mammalian (or neocortical) brain regions (MacLean, 1990). In this didactic simplification, the reptilian brain hosts neural systems of ultimate importance for unconscious autonomous bodily functions (e.g., breathing and heart beat regulation) and very ancient emotions such as FEAR, LUST, RAGE, and SEEKING, while the limbic brain, more developed in mammals than reptiles, not only adds additional complexities to those most ancient emotions, but also forged neural circuits for higher social emotions such as maternal CARE, separation-distress (PANIC) as well as social PLAY, all of which will be described in this section. The most recent development in mammalian, especially primate, brain-mind evolution led to a “cortical thinking cap”, most expansive in humans (at least as percent of body weight), enabling us to reason, but also to cognitively regulate our emotions. Since the primal emotions are located in the two phylogenetically oldest layers of reptilian and mammalian brains, individual differences in these ancient neural circuitry represent, from our perspective, the primal foundations of the major affective human personality dimensions, as well as those of other animals. Of course, a great deal of cortical-cognitive competence is permitted by neo-cortical regions, but it has long been known that upper brain-mind regions can not operate effectively without the genetically dictated subcortical emotional-affective, motivation and consciousness sustaining systems.

Until now, seven primal emotions have been identified by Panksepp (1998, 2011—largely using deep brain stimulation approach), which all could be of relevance to understand human personality. Among these are four emotional circuitries for positive emotions (SEEKING, LUST, CARE, PLAY) and three emotional circuitries for negative emotions (FEAR, RAGE/ANGER and SADNESS/PANIC). By means of electrical stimulation of the mammalian brain and also psychopharmacological challenge tests, many investigators of brain-behavior-relationships have demonstrated that these emotional brain systems have been highly conserved across mammalian species, thereby making it likely that our fellow mammalian animals also probably exhibit comparable personality dimensions. Supporting this idea, most of the personality traits represented in the Five Factor Model of Personality have been observed in mammals, with dogs having been especially well studied (Gosling and John, 1999; Gosling et al., 2003), although Conscientiousness has been effectively studied only in humans and chimpanzees.

Before proceeding, we would briefly discuss the primal emotions identified through a cross-species study of subcortically situated emotional systems. The SEEKING system is our general purpose appetitive-exploratory-investigatory system, that is essential for acquiring all the environmental resources needed for survival and propagation. The LUST and CARE urges are deeply entwined with SEEKING circuitry—thereby promoting sexual desire and the care of offspring. LUST, in combination with SEEKING urges, promotes sexual engagements. When the CARE circuit is aroused, humans as well as other animals feel an urge to nurture and protect their offspring. Arousal of this circuit may also promote a satisfying relationship among adult partners.

Of course, young animals must also have systems that help them prepare for their adult emotional activities. Social PLAY circuitry, also closely related to SEEKING urges, is of such evolutionary importance: Without abundant pro-social activities during development, as promoted by PLAY urges, children may have diminished social competence in adulthood (Pellegrini and Smith, 1998). Indeed, animal models support the idea that a lack of early PLAY during childhood might result in ADHD tendencies (Panksepp et al., 2003). Parenthetically, the massive elevation of non-social SEEKING, and diminished social PLAY, may create future social problems unique to emerging human *digital-societies*: Around the globe children are spending excessive time on smartphones, tablets, and computer screens, which reduces natural social PLAY time. The diminished opportunities for rough and tumble PLAY activities may have unforeseen social consequences (see Montag et al., 2016b).

Activities in the various primary positive and negative emotional systems are accompanied by distinct affective feeling states, which presumably promote different learned behavior patterns. We now switch to the negative brain emotional networks. Activation of the neural FEAR circuitry signals danger and helps us escape diverse, dangerous life-threatening situations. RAGE helps us defend our lives and other resources—but in complex human societies, with cultural expectations about what is deemed proper behavior, can obviously backfire. RAGE is not only activated when we are attacked and need to defend ourselves, but also in situations of frustration, when access to expected reward is thwarted, including territorial conflicts. Finally, the PANIC/SADNESS circuitry is activated in situations of separation distress going along with feelings of loneliness and sadness, which may eventually precipitate depression. From an evolutionary perspective this makes sense, as activity in the SADNESS circuit (as well as the other negative circuitries) is accompanied by negative feelings. Humans strive to reduce activity in these circuitries, because they simply feel awful to varying degrees depending on the environmental situations triggering the various emotional systems: From an evolutionary perspective, feeling bad when left alone or losing a loved person is an evolutionary advantageous response – in social species, especially when individuals are very young, being alone can be very dangerous. This ancestral survival signal that we typically call the PANIC system (as mapped out with deep brain stimulation) was important not only among our ancestors, but survival and thriving in our modern complex environment often requires social bonds that promote cooperation that can enable social alliances. When one is alone, whether mouse or man, it is simply more difficult to survive, and to get through life with an affectively positive frame of mind. Thus *homo sapiens*, like all other mammals, are genetically “social animals” who actively SEEK companionship. Therefore SEEKING and SADNESS activities are strongly linked to each other. In depression SEEKING activity is downregulated (the patient has no motivation/energy) and the SADNESS circuitry shows full blown activation (see also Panksepp, 2015b, 2016; Montag et al., 2016c).

## On the Relationship of Motivational and Emotional Systems

Although the behavioral and psychological sciences have often distinguished “motivational” and “emotional” processes, they are highly overlapping concepts. The basic brain motivational systems often include homeostatic processes, such as maintenance of bodily energy (hunger), water (thirst) and other states essential for survival, from thermal homeostasis to micronutrients such as sodium. It is important to highlight that such basic motivational systems may have dedicated need-state detectors in the brain and body, but they do not have separate “motivational systems” to acquire the needed resources.

All psychologically salient bodily need states operate through the auspices of a general purpose SEEKING system—which is still often called “The Brain Reward System.” In fact, this “self-stimulation” circuitry does not directly detect any of the specific substances needed for survival, but all the need detectors of the body and brain operate through a general purpose SEEKING system that motivates general appetitive behaviors (from exploration to specific learning-direct seeking of specific resources). In the present context, it is important to note that there are strong evolutionary linkages between such need detector systems in all animals (with species-typical variations), but until recently none of these systems have figured explicit in human personality theory. On the other hand, the general purpose SEEKING urge, which promotes a large variety of goal-directed behaviors, is of great importance for personality traits such as eagerness and enthusiasm.

One might also be tempted to ask “Are motivational or emotional systems evolutionary older?”. Again, this is not a productive way to proceed. All emotional systems have strong motivational components. And systems designed to protect the homeostatic integrity of our bodies (body-need detectors) operate through the emotional/motivational SEEKING system. Before neuroscience, psychology was accustomed to focussing mainly on “conceptual kinds.” With advances in our undestanding of the relevant evolved, subcortical “survival systems” [which come in emotional, homeostatic, and sensory (also interoceptive) varieties], we are now in a better position to identify which are most important for the emergence of a neurobiological understanding of personality. In our estimation, the ancestral *emotional* systems are most important for guiding us toward a naturalistic understanding of human and animal personalities, especially since they now allow a variety of novel neurobiological predictions as opposed to just providing cognitive-psychological-dimensional views of personality. We think that the ever-popular *Five Factor Model of Personality* currently needs some primary-process emotional grounding in order to give a better idea of what those factors may reflect at fundamental affective levels, which may provide better neural understandings of the emotional foundations of human personality.

## Linking Primal Emotions to the Five Factor Model of Personality

The Five Factor Model of Personality arguably represents one of the most important personality models currently used world-wide in both research and applied work settings (e.g., Barrick and Mount, 1991; Costa and McCrae, 1992; McCrae and John, 1992; McCrae and Costa, 2008). It has been based on lexical perspectives in the early psychological sciences (Allport and Odbert, 1936; Cattell, 1943; Fiske, 1949), where psychologists in the early 1930s/40s started to study human language by means of factor analysis resulting in five personality dimensions easily to be remembered with the acronym **OCEAN**: ***O****penness to Experience* describes humans who are open to new experiences (for example this could be in the domains of food, traveling, culture), intellectual and artistic pursuits which surely would contribute to a sense of aesthetics. ***C****onscientiousness* describes diligent and punctual persons, who can be relied upon. ***E****xtraverted* persons are assessed on dimensions of socially outgoingness and assertiveness. ***A****greeable* persons are team-players and associated with higher empathic traits (see also Melchers et al., 2016). Finally, ***N****euroticism* can be characterized by being moody, emotionally unstable and anxious, with tendencies toward depressed states.

The logic behind the lexical approach follows the convincing idea that personality should manifest itself in human language, which we use on a daily basis to describe ourselves and others. Hence, the dissection of human language use with statistical tools should reveal something about our fundamental psychological personality traits. For instance, a person describing his or herself as cold, unkind, selfish and uncooperative could be described by a higher order factor of (low) *Agreeableness* (Goldberg, 1992). Of note (and in contrast to older typologies in personality; see the approach by Galen as mentioned by Stelmack and Stalikas, 1991) every person has a score on each of the already introduced personality dimensions/traits resulting in a personality pattern more or less unique to him/her. Humans differ on personality continuums, which means that a person is not either extraverted or introverted, but tends more in the one or the other direction of the investigated continuum, often based on environmental affordances (see also the discussion above on the stability of personality across different situations).

The literature has also coined the cluster of OCEAN personality dimensions as the *Big Five of Personality*. These factors have been observed to appear relatively stable across different cultures (McCrae et al., 1998; McCrae and Allik, 2002). Of note, the Big Five and the Five Factor Model of Personality are historically *not* exactly the same: The Big Five hint more toward the original *lexical analysis* approach (see also Goldberg, 1992). In contrast, the Five Factor Model refers to a more explicit *model of personality*, although based on the history of the lexical analysis approach, using formulated items instead of adjectives to assess individual differences in these personality traits (see NEO-FFI or NEO-PI-R by McCrae and Costa, 2004). Given the overlap in the naming of the dimensions and also in the concepts underlying these traits, in our opinion Big Five/Five Factor Model of Personality can typically be used interchangably (as is commonly done in many works).

Before we describe how the Big Five and the primary emotional systems may be linked to each other, we would note that there is currently no general consensus on how many personality dimensions are needed to fully grasp human personality. Besides the Big Five, many other (indeed various biological) personality theories exist: For instance there are (i) three dimensions in Eysenck’s PEN model (Psychoticism, Extraversion and Neuroticism; Eysenck et al., 1992), (ii) three dimensions in Gray’s revised Reinforcement Sensitivity Theory (Gray and McNaughton, 2000; Leue and Beauducel, 2008; Markett et al., 2014; Reuter et al., 2015) and (iii) four temperament- and three character-traits in Cloninger’s extensive model (Cloninger et al., 1993). Also, Cloninger’s model splits personality into temperament and character. According to Cloninger, temperament describes highly inherited personality traits visible already in infants (clearly also being linked to primal emotions), while character traits are shaped more by the environment and one’s own unique learning-history as stabilized in early adulthood.

Costa and McCrae (1993) came up with the metaphor that the Five Factor Model of Personality somehow resembles the Christmas tree on which each of the other personality models can serve as “decorations.” Indeed, this seems reasonable to the extent that the aforementioned personality traits from other theories can often be related to one or two dimensions of the Five Factor Model of Personality. With respect to primal emotional systems, we would envision such a tree metaphor rather differently. Although links between individual differences in primal emotions and the Big Five exist (see next paragraph), we currently have a much better neurobiological understanding of the primal emotions than the nature of the aforementioned “tree.” This point may become better understandable if we recall that our key argument is that the primal emotions represent the phylogenetically oldest part of human personality, with that foundation driving behavior and the more cognitive aspects of personality via a bottom–up affective developmental-learning trajectory (Panksepp, 1998). This foundational perspective may be able to link up with cross-species neurobiological analyses more than top–down human-centric cognitive-linguistic views.

In the early work of Davis et al. (2003) individual emotional differences as assessed by the *Affective Neuroscience Personality Scales* (ANPS) have, for the first time, provided a cross-species neuropsychological context that can neuropsychologically illuminate the Big Five (see **Table 1**). The ANPS has been constructed on the theoretical background of Panksepp’s neuro-evolutionary, cross-mammalian *Affective Neuroscience* (AN) theory assessing, via self-report, individual differences in SEEKING, CARE, PLAY (positive emotions) and FEAR, ANGER, SADNESS (negative emotions)^1^. Each of these emotions represents a robust, evidence-based neuro-behavioral-psychological *tool for survival* driving our actions in a bottom–up fashion (see introduction above). Usually our cortical thinking cap holds a tight grip over the emotional activity in these ancient circuitries (please note, that following radical neodecortication, animals exhibit hyper-emotionality, as in decorticate-rage). In short, all the basic emotions survive radical decortication of infant animals (e.g., Panksepp et al., 1994). Therefore, pure raw affects rather seldom dominate everyday human life. Nevertheless, everyone knows how it feels, when our strongly driven genetic primary emotional programs are working at full intensity, often overwhelming us (remember situations of being profoundly terrified at the sight of danger or purely happy when playing with someone). The ANPS has been validated in a number of studies in the last few years using different neuroscientific methods. Among others, an inverse correlation between the ANGER dimension and left amygdala volume has been demonstrated (Reuter et al., 2009). While Felten et al. (2011) demonstrated an interaction effect of dopaminergic polymorphisms on SADNESS, Montag et al. (2011) showed an interaction between serotonergic and oxytocinergic polymorphisms on SADNESS and FEAR. Recent work by van der Westhuizen and Solms (2015) investigated associations between dominance and the ANPS, whereas also the testosterone to cortisol ratio was positively associated with dominance, but with none of the ANPS dimensions. A recent resting state fMRI study by Deris et al. (2017) observed that functional connectivity of the basolateral amygdala (here the seed was planted) to other brain regions can be linked to the primal emotion of SADNESS. A study by Sindermann et al. (2016) showed an association between the 2D:4D marker of the hand (an indice for prenatal testosterone exposure) and FEAR/SADNESS in female participants. Recent works relying “only” on self-report ANPS questionnaire data investigated a link between SEEKING and depression in stroke patients (Farinelli et al., 2013), while others have used the ANPS in the context of personality disorders (Geir et al., 2014; Karterud et al., 2016) and depression patients (Montag et al., 2016c). The interest of many researchers in the ANPS mirrors also in many available translations such as Turkish (Özkarar-Gradwohl et al., 2014), Spanish (Abella et al., 2011), French (Pahlavan et al., 2008; Pingault et al., 2012), and German (Reuter et al., 2017).

**Table 1:** Associations between the Big Five (assessed with Goldberg’s inventory) and dimensions of the ANPS in an **US** sample taken from Davis et al. (2003).

	SEEKING	FEAR	CARE	ANGER	PLAY	SADNESS
Neuroticism	-0.01	**0.75**	0.07	**0.65**	-0.12	**0.68**
	n.s.	**<0.001**	n.s.	**<0.001**	n.s.	**<0.001**
Extraversion	0.13	-0.19	0.25	-0.04	**0.46**	-0.21
	n.s.	<0.05	<0.01	n.s.	**<0.001**	<0.01
Openness	**0.47**	-0.05	0.06	-0.08	0.13	-0.00
	**<0.001**	n.s.	n.s.	n.s.	n.s.	n.s.
Agreeableness	-0.01	-0.17	**0.50**	**-0.48**	0.29	-0.13
	n.s.	<0.05	**<0.001**	**<0.001**	<0.001	n.s.
Conscientiousness	-0.01	-0.24	0.12	-0.30	0.00	-0.30
	n.s.	<0.01	n.s.	<0.001	n.s.	<0.001


*Note that correlations of >0.45 have been indicated by bold letters in the original publication by Davis et al. (2003). Moreover, for better comparability with **Tables 2**, **3** we changed the terms *Emotional Stability* to *Neuroticism* and switched the algebraic signs accordingly. Student sample: *n* = 171 (50 males, 121 females).*

**FIGURE 1:**
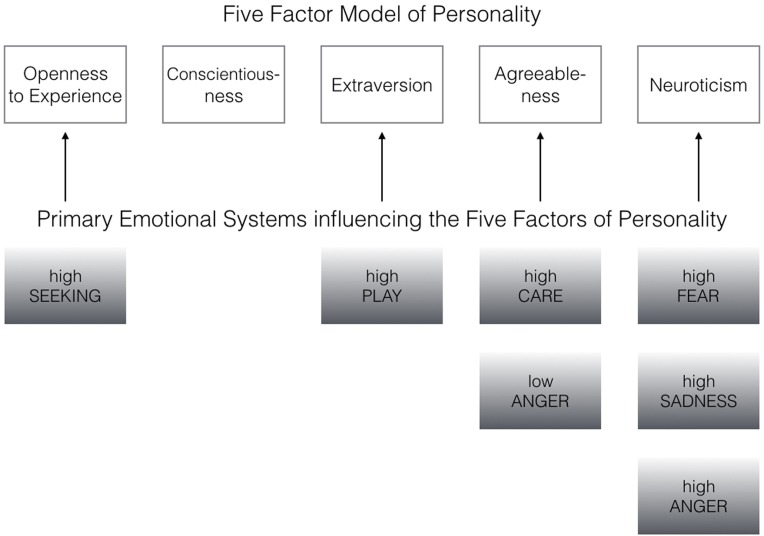
**Primary emotional systems influencing the Big Five of Personality bottom–up (the associations have been derived from the most robust correlation patterns as depicted in **Tables 1–3**)**.

Coming back to the original ANPS work by Davis et al. (2003), but also the revised newer work by Davis and Panksepp (2011), we see some robust correlations between the Big Five and primal emotions. Arising from the correlation patterns in the original works, SEEKING is robustly linked with Openness to Experience and high PLAY goes along with higher Extraversion. High CARE and low ANGER are associated with higher Agreeableness. High scores on all negative emotional circuitries might underlie Neuroticism (with strongest effects for SADNESS and FEAR). As these associations have been derived from correlational studies, causality on our bottom–up idea of primary emotional systems driving human behavior/personality cannot be simply inferred from these psychological data. Considering the architecture of the human brain, locating primary emotional systems in the phylogenetically oldest part of the mammalian brain, it is very likely and logical that individual differences in these circuitries represent the oldest part of personality and influence complex personality traits such as the Big Five in a bottom–up fashion. This idea becomes even more convincing, as we consider the patterns of our cross-cultural data (in **Tables 1–3**): The same associations between the Big Five/Five Factor Model^2^ and primal emotional systems in three samples from different cultural backgrounds can be observed, which speaks for a global ancestral neuro-biological effect, because the cultural environments differ strongly in the USA, Germany, and China. Further evidence for the global robustness of our findings comes from the fact that the samples did not only differ in terms of ethnic background, but also the observed gender ratios and age. Whereas the sample from China consists of more male than female participants, the samples from Germany and the USA consist of more female than male participants. This can be explained by the fact that the recruiting process in China was conducted at a technical university with a much higher proportion of male students, whereas in Germany and USA among others psychology students were recruited with a higher female ratio. Again, given the heterogeneity of the samples with respect to culture, gender-ratio and differences in age, quite similar correlation patterns (as summarized in **Tables 1–3**) strongly point toward global trends (note that the bold printed results are robust and would hold for multiple testing, if we consider to replicate the seven main correlation depicted in **Tables 1–3**; Bonferroni correction for seven tests results in alphas of 0.05/7 = 0.007). Therefore, we summarize the findings of these global trends also in **Figure 1** and depict which primary emotional systems underlie each of the Big Five of Personality. Although Conscientiousness can be predicted from some primary emotions (in some countries), the associations are weaker and less stable across the samples. Moreover, Conscientiousness cannot be observed in most mammals, therefore we refrain from the inclusion of potential underlying primal emotions in **Figure 1**.

**Table 2:** Associations between the Five Factor Model of Personality (assessed with the NEO-FFI) and dimensions of the ANPS in a *German* sample.

	SEEKING	FEAR	CARE	ANGER	PLAY	SADNESS
Neuroticism	-0.255	**0.752**	0.135	**0.328**	-0.363	**0.689**
	<0.001	**<0.001**	<0.001	**<0.001**	<0.001	**<0.001**
	[-0.336; -0.164]	[0.716; 0.784]	[0.049; 0.215]	[0.255; 0.397]	[-0.429; -0.290]	[0.650; 0.728]
Extraversion	0.358	-0.391	0.227	-0.155	**0.668**	-0.305
	<0.001	<0.001	<0.001	<0.001	**<0.001**	<0.001
	[0.272; 0.430]	[-0.454; -0.323]	[0.148; 0.309]	[-0.238; -0.076]	[0.621; 0.709]	[-0.366; -0.244]
Openness	**0.388**	-0.015	0.242	-0.019	0.041	0.157
	**<0.001**	0.688	<0.001	0.613	0.284	<0.001
	[0.308; 0.457]	[-0.096; 0.067]	[0.165; 0.315]	[-0.097; 0.061]	[-0.039; 0.125]	[0.080; 0.234]
Agreeableness	0.152	-0.065	**0.488**	**-0.405**	0.331	0.010
	<0.001	0.090	**<0.001**	**<0.001**	<0.001	0.798
	[0.063; 0.237]	[-0.147; 0.022]	[0.414; 0.558]	[-0.477; -0.331]	[0.255; 0.403]	[-0.064; 0.092]
Conscientiousness	0.268	-0.029	0.097	-0.023	-0.006	-0.027
	<0.001	0.454	0.011	0.541	0.869	0.483
	[0.196; 0.342]	[-0.115; 0.059]	[0.012; 0.176]	[-0.100; 0.061]	[-0.086; 0.069]	[-0.113; 0.058]


*In bold letters we highlight the correlations of largest interest from the Davis et al. (2003) study. Comparable correlation patterns as in **Table 1** can be observed with the highest difference in the lower ANGER – Neuroticism association. *n* = 687 [212 males, 475 females; mean-age = 23.64 (*SD* = 6.00)], mainly students. Confidence intervals (CIs) were calculated using bootstrap analysis (1000 samples, Bias corrected and accelerated).*

**Table 3:** Associations between the Five Factor Model of Personality (assessed with the NEO-FFI) and dimensions of the ANPS in a *Chinese* sample.

	SEEKING	FEAR	CARE	ANGER	PLAY	SADNESS
Neuroticism	-0.234	**0.725**	-0.095	**0.401**	-0.266	**0.594**
	<0.001	**<0.001**	0.059	** <0.001**	<0.001	** <0.001**
	[-0.359; -0.110]	[0.670; 0.772]	[-0.220; 0.037]	[0.296; 0.492]	[-0.370; -0.163]	[0.523; 0.658]
Extraversion	0.349	-0.461	0.353	-0.197	**0.587**	-0.263
	<0.001	<0.001	<0.001	<0.001	** <0.001**	<0.001
	[0.231; 0.461]	[-0.548; -0.368]	[0.241; 0.451]	[-0.308; -0.078]	[0.506; 0.658]	[-0.361; -0.156]
Openness	**0.395**	-0.013	0.241	0.011	0.189	0.073
	**<0.001**	0.790	<0.001	0.827	<0.001	0.147
	[0.282; 0.493]	[-0.110; 0.083]	[0.137; 0.347]	[-0.126; 0.137]	[0.088; 0.289]	[-0.027; 0.182]
Agreeableness	0.148	-0.273	**0.382**	**-0.401**	0.336	-0.228
	0.003	<0.001	** <0.001**	**<0.001**	<0.001	<0.001
	[0.021; 0.287]	[-0.385; -0.142]	[0.277; 0.482]	[-0.481; -0.318]	[0.234; 0.430]	[-0.327; -0.121]
Conscientiousness	0.381	-0.271	0.162	-0.086	0.119	-0.203
	<0.001	<0.001	0.001	0.087	0.018	<0.001
	[0.261; 0.492]	[-0.381; -0.158]	[0.041; 0.290]	[-0.196; 0.038]	[0.010; 0.234]	[-0.314; -0.086]


*In bold letters we highlight the correlations of largest interest from the Davis et al. (2003) study. Comparable correlation patterns as in **Table 1** can be observed with the highest difference in the lower ANGER – Neuroticism association. *n* = 394, [263 = males, 131 females; mean-age = 21.91 (*SD* = 2.35)], mainly students. Confidence intervals (CIs) were calculated using bootstrap analysis (1000 samples, Bias corrected and accelerated).*

## Toward A Modern Understanding of Human Personality

**FIGURE 2:**
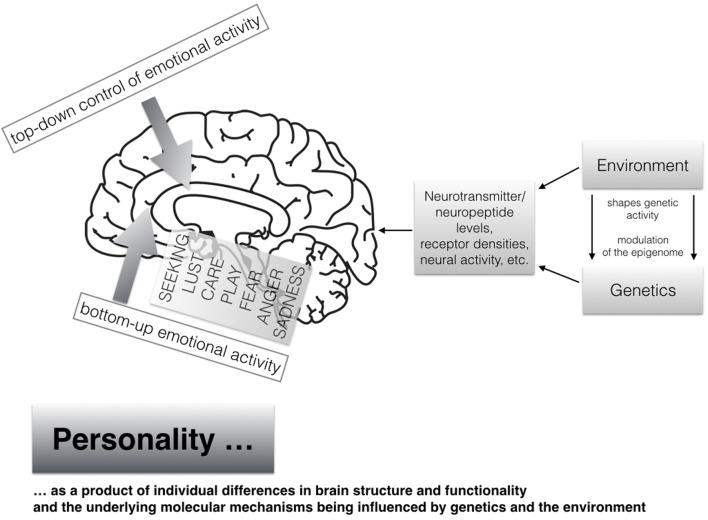
**A modern view on human personality arising from individual differences in brain structure and functionality shaped by both genetics and the environment (the brain image has been taken from Pixabay.net and is license free); moreover individual differences in bottom–up-emotional-bursts in a concert with individual differences of top–down-regulation capacitites result in unique personality patterns**.

At the end of this section, we will explain our current understanding of the neuropsychological sources of human personality as shown in **Figure 2**. It is common sense that individual differences in personality are strongly influenced by both genetics as well as diverse environmental events and experiences [see mega-analysis by Polderman et al. (2015) which studied more than 14 million twin pairs]. Heritability estimates come up with about 50% of genetic influences and 50% of the environment (as very rough estimates), which also has been supported with comparable numbers (on average) with respect to the here discussed primal emotional systems as assessed with the ANPS in a recent study (Montag et al., 2016a). It is noteworthy that twin studies are only able to estimate the genetic and environment effects of *individual differences* in personality. Given the strong homologies that exist in the neuroanatomical and neurochemical foundations of the primary emotional systems across different mammalian species, the neuro-genetic effects beyond those already described in twin studies must be very strong. It is of importance to further note that genetics and environmental influences cannot be seen as distinct entities, because both interact and shape together what is happening on both molecular and molar levels, in both brain and mind, resulting in individual differences in brain structure and functionality. This in turn gives way to individual differences in human personality, which are usually studied linguistically. The study of the modulation of the so called epigenome, by including environmental influences, will surely bring new insights also to personality research in the coming years (e.g., see how prosocial personality is linked to different methylation patterns of the gene coding for oxytocin; Haas et al., 2016). In short: the epigenome represents the area “directly above” the genetic code—namely how the environment modifies long-term gene-expression patterns in all of our cells, most importantly neurons from the present perspective. Given the representation of the vast human genome in every cell nucleus, much of the human genome is densely packed and therefore not accessible at all times. To oversimplify, the modulation of the epigenome opens or closes the genome, so that various regions of animal bodies are able to get differential (environmentally controlled) access to information about the blueprints of any of the molecules that are needed to build the kind of creatures that we are (e.g., McGowan and Szyf, 2010; Heim and Binder, 2012; Toyokawa et al., 2012).

Many of the epigenetic mechanisms are still not well understood, but it is generally agreed that bringing the study of the epigenome to personality research (and its phylogenetically old component primal emotions) will be an important step for a better understanding of human personalities—in a sense, a deeper understanding of who we are. This further strengthens the dictum that we must better envision how both nature and nurture, as interacting partners rather than distinct units, create our various psychological strengths and weaknesses. Understandably, this exciting area of research is now rapidly expanding. We would only note that the interaction of genetics and environment was already nicely described with early prominent results from Caspi et al. (2003) describing how the risk to suffer from depression is elevated in persons who carry both a genetic risk factor for depression and the vicissitudes of a severe, adverse life event in childhood (for a meta-analysis of this topic, see Karg et al., 2011). Clearly it is also of importance to mention studies, which highlight exclusively the impact of environmental influences (in the following mostly traumatic experiences) on one’s own activity of primary emotional systems manifesting in diverse areas such as one’s own mimicry and/or vagal regulation [investigated in street boys by Ardizzi et al. (2013) and in maltreated children by Ardizzi et al. (2016)], emotion recognition abilities/selective attention when processing facial emotion (Pollak and Sinha, 2002; Pollak and Tolley-Schell, 2003; Pollak et al., 2009; Scrimin et al., 2009), denial of emotions such as sadness (in terms of lower recognition rates of sadness in others when being a survivor of war in Sierra Leone; Umilta et al., 2013) or interoceptive sensitivity (Ferri et al., 2013). In sum, many studies investigated main or epistasis effects of molecular genetic markers on temperament traits being closely linked to primary emotional systems (e.g., Montag et al., 2010a,b), main effects of environmental factors as mentioned in this section and most importantly the interaction effects between genetics and the environment.

To return to the importance of the study of primal emotional systems for understanding complex human personality traits such as the Big Five, we would simply note that the unique personality of a person will be shaped by the strength of both the tonic/phasic emotional bursts from the subcortical levels of the brain, together with one’s ability to hold a tight grip on the emotional activities (abilities in emotion regulation) – hence cortical, top–down regulation. Unique patterns of individual differences in both bottom–up motivational energy derived from primary emotional systems together with individual differences in top–down regulation capacities from higher cortical brain regions, remain important keys to understand the developmental creation and molding of human personalities. As an example one could understand the angry personality type as a result of tonic strong bursts from the ancient bottom–up RAGE circuitries together with diminished cortical top–down control (compare this with Siever’s, 2008 view).

## A Closer Look At Personality From An Evolutionary Perspective

The following section summarizes some critical points of Nettle’s theory on why variation in personality still occurs. Going beyond his thoughts we will add additional layers, especially primal emotional systems as *tools for survival* to his model. Nettle’s theory (Nettle, 2006) is strongly built on Darwin’s idea of *natural selection* (Darwin, 1859/2008), which Charles Darwin among others (and also Nettle, 2009; p. 54–78) illustrated with the famous Galapagos finches (see also Darwin’s Beagle diaries for his first mentions of the finches; Costa and McCrae, 1994). On various Galapagos islands Darwin observed finches with different formed beaks. Until our days there are large differences in beak size to be registered between the finch populations of the different Galapagos islands, but also variation of beak size on each island is evident. This phenomenon can be explained by a principle called *fluctuation selection*, ultimately helping a species (seen as a whole) to survive in different environments. A key to understand the different sizes of the finches’ beaks is the insightful idea that the islands of Galapagos (and niches on the islands) differ in what they offer in terms of food availability for the finches. On islands and their niches with large and hard to crack seeds those finches with large beaks would survive best, producing the most offspring, because large beaks facilitate the cracking of large seeds. On islands with small/soft seeds or worms to be retrieved from crevices, finer beaks would be of advantage, because large beaks would hinder getting at the smaller and more hidden natural resources. Hence, on islands with smaller seeds and worms, finches with smaller beaks would be better able to find food and as a consequence produce more offspring (see for additional new insights the work by De León et al., 2014). Although this all is very logical, many critical questions remain: If on one island large beaks seem to be better and small beaks on others, why is there still some variation in the size of the large/small beaks on each island? *Natural selection* could easily have chosen a winning solution on every island, favoring ultimately and for all times large or small beaks. A key to understand the variation remaining on each island has been addressed by the so called *trade-off model*. A trait such as a small/large beak comes with advantages and disadvantages (as we have seen each beak is only optimal for retrieval of certain foods). What is to one’s advantage in a finch population at a given time, can change rapidly with weather fluctuations. It has been shown that changes in the climate of an island can change the availability of certain kinds of food. Therefore, in times of a drought, an island, which beforehand may have advantaged finches with small beaks (with plenty availability of small/soft seeds), may now make food more available for birds with larger beaks (as only large hard seeds remain as food; see Grant and Grant, 1989)^3^. For example, due to droughts and the resulting diminished availability of certain foods, finches with the larger beaks from the beak population may have better chances for survivial, because they can now better access the available food: “This selection event was followed by another favoring birds with deep beaks capable of extracting the only available food, arthropods from beneath the bark of trees and within Opuntia trunks and old pads.” (Grant and Grant, 1989, p. 377). Variation in the beak size on each island’s finch population therefore promoted the overall survival of sub-species with certain characteristics. The term *fluctuation selection* (see also Nettle, 2009, p. 64) illustrates that not always is the same trait associated with higher survival, but rather that the trait that is best adapted varies with environmental changes.

As Nettle postulates, this idea can be transfered to human personality. Different kinds of personality traits come with different advantages or disadvantages. Again, this ultimately depends on the kind of natural and social environment we are dealing with. When *homo sapiens* started to populate (and “conquer”) the world out of Africa about 60–70,000 years ago, they were facing different environments in terms of fauna and climate. Therefore, variation in traits such as personality may have helped our species to better adjust to different environments, both physical as well as social. To illustrate this, let us choose an example with neurotic vs. emotional stable or extraverted vs. introverted persons. In dangerous environments the neurotic person will have an advantage, because he/she is more careful in monitoring the (social) environment and as a consequence will face less often immediate dangers compared with an emotional stable person (ergo survival is more likely in neurotics; see also Denissen and Penke, 2008a,b). In contrast, when the environment is safe, the neurotic person may spend personal emotional resources on monitoring a safe environment which might be better invested in the search for food or a mating partner. Here, the emotional stable, but perhaps also the extraverted person, has an advantage. This specific possibility is nicely supported by empirical data showing that extraverts have more children and more mating partners, but are also more prone to end up in the hospital due to accidents (Nettle, 2005; see for more recent literature in this area the overview provided by Penke and Jokela, 2016). In Table 1 on page 628 of Nettle’s paper from 2006, such trade-offs for each personality trait out of the Big Five of Personality approach are described. We briefly summarize them here: Being extraverted comes with advantages in mating success and building social alliances, but also potential disadvantages such as taking higher physical risk accompanied perhaps by less family stability. Open persons are more creative with putative positive effects on their attractiveness, but people who are extremely high on the Openness dimension may be more prone to psychosis or unusual beliefs. Agreeable persons come with the advantage of having positive and functional relationships, but they often can be exploited by others for their high empathy and goodness. Hence they may not be able to maximize personal profits as low agreeable persons might do (for an example from agreeableness and stuttering, see Jafari et al., 2015). Neuroticism comes along with higher attention/monitoring of potential dangerous situations, but also higher proneness for affective disorders. Finally, high Conscientiousness has long term benefits in the area of health behavior and longevity, but conscientious persons might also become overly obsessed with details, thereby miss some immediate easy access to available reward.

We argue, beyond the ideas advanced by Nettle (2006, 2009), to also consider variation in primal emotions to better understand the summarized trade-off models, because (i) such states of mind have been strongly shaped by evolution and we have argued here (ii) that affective strengths and weaknesses are essential parts of human personality. Therefore trade-offs arising from different strengths of bottom–up operating primal emotions and top–down cognitive strategies might be important to understand the postulated trade-offs by Nettle for the Big Five personality dimensions.

We summarized in **Table 4** why evolution may have favored the solution of seven inbuilt primal emotional systems in our mammalian brains. Beyond the evolutionary necessicity of each primal emotional system, it is also noteworty that individual differences in brain structure and function of these emotional circuits could result in different strengths/activities with which each primal emotional system usually operates. The above mentioned example of the neurotic person can be well described by a more arousable FEAR system which may drive neurotic behaviors (see **Figure 1**). The extraverted person might be pushed toward one side of the trade-off due to a high PLAY system and so forth.

**Table 4:** Primary emotional systems as tools for survival.

Primary emotion	Evolutionary tool for…
SEEKING (+)	The SEEKING system provides mammals with psychological “energy” (i.e., enthusiasm) to explore the environment. This is necessary to find mating partners as well as food to nourish both brain and body.
LUST (+)	LUST activity is of importance to be attracted to the opposite sex and transfer one’s own genome (hence also of species *homo sapiens*) in terms of offspring to the next generation.
CARE (+)	Humans are social mammals. In social groups survival chances are higher. Moreover, taking CARE of one’s own offspring helps assure that the young children grow into adults and themselves can have families.
PLAY (+)	PLAY behavior is of importance to learn social competencies and motoric skills. This helps to get better along in complex social groups when being an adult.
FEAR (-)	Without a FEAR response (along with the learning it promotes) homo sapiens would not have optimal abilities to escape and avoid dangerous situations and to carefully monitor the safety of their environments.
RAGE/ANGER (-)	Activity of the RAGE system is observed when mammals are in need to defend themselves (when a predator is closing in), but also in situations of frustration, when an expected reward is absent. RAGE activity is also visible to solve territorial conflicts in mammals.
SADNESS/PANIC (-)	PANIC/SADNESS reflects separation distress and signals a situation of having lost contact with an important person or being lost in the environment. As homo sapiens is a social animal, separation from a caregiver or another important person triggers a distress reaction leading to distress vocalization (crying) to reunite with a partner or a parent. Ultimately, as with CARE, *homo sapiens* is stronger in groups than when alone. CARE activities also can counteract and downregulate SADNESS arousal.


*(+) positive primal emotions; (-) negative primal emotions.*

But again, variations of primary emotional systems underlying individual differences in personality are important from an evolutionary perspective: For instance, not all constellations of primal emotional systems operate successfully in a given environment. Someone with a high active LUST system might use the energy spend on the arousal of this system better in CAREing for his/her already existing offspring instead of searching for an extramarital affair (in this example we might also note how SEEKING arousal might be hijacked by the LUST circuitry). On a species level “different fitness optima of heritable variants over time and space can maintain the heritable variation” (Buss, 2009, p. 362), hence it secures survival of a species. On an individual level the situation could be a bit different. Although the average fitness of the normal range of personality could be equal (perhaps with the exception of the distribution extremes, which might cause psychopathologies), each unique personality might differ in their success to reach one’s own optimal fitness given a non-optimal fit with a given environment (see als Nettle, 2006, p. 623). Therefore, all human beings need to find the right social-environmental niches, where their intrinsic personality variability, including the underlying primal emotions, operate “best” (i.e., promote survival and thriving).

In sum, we believe some of the major driving forces of Nettle’s trade-off personality model can be related directly to strengths and weakness in the underlying primal emotions, as they are “survival systems” constructed by a long evolutionary selection process. Their fundamental structures were genetically constructed (they are in-built unconditional affective-behavioral networks within our brains). Variation of these systems across species can enlarge or diminish the chances of survival of species in specific environments (again, we are not speaking about primary emotional systems being there or absent in terms of absolute (1 or 0) function).

## Outlook: The Study of Individual Differences in Raw Affect in Humans

Summing up the arguments from this essay, we hope that many readers will be encouraged to increasingly include measures of primal emotional systems (aside from just the more established personality measures) in their studies so as to better understand human personality, namely in ways that can be connected to neuroscientific issues. This can be achieved via many routes. The easiest way to follow for many personality psychologists represents the inclusion of the *ANPS* in their studies (Davis et al., 2003; Davis and Panksepp, 2011) so as to have a self-report measure which has been derived from rich evolutionary and neuroscientific perspectives—a tool that has undergone several validation studies in the past few years (e.g., Reuter et al., 2009; Montag et al., 2016a,b; Sindermann et al., 2016).

The fact that the ANPS has been constructed on a neuroscientific background brings along several advantages compared to those based simply on lexically derived measures. For biologically oriented psychologists, the use of these measures holds the chance to indirectly hypothesize about the neuroanatomical areas and brain chemistries of relevance for various personality styles. For applicability see some recent works in the context of facial expressions by Montag and Panksepp (2016) or Internet addiction (Montag et al., 2016b). To illustrate this briefly: The work of investigators interested in the study of the biological basis of Neuroticism may be facilitated if they better understand that the brain systems that may drive neurotic behavior bottom–up, do this via FEAR, PANIC-SADNESS (and to a lesser degree RAGE-ANGER) systems. Both activity in the FEAR and SADNESS circuits can be downregulated by brain opioids and oxytocin (Panksepp, 1992, 1998; Kirsch et al., 2005). Therefore, the study of the neuropeptide oxytocin and various endogenous opioids might be good key candidates to be considered in such a research endeavor (e.g., with the prediction that lower brain oxytocin and opioid levels may be associated with higher anxiety, especially separation-anxiety?).

Nevertheless (and needless to say), self-report measures are confounded by top–down brain activity via diverse tertiary-process level from cognitive to cultural styles – namely our cortical thinking cap, which can inhibit primal subcortical emotional powers, at times may regulate and thereby modify (e.g., hold a tight grip on) our emotions. Furthermore, verbal reports are only an indirect path to the study of one’s own emotionality. Davis and Panksepp (2011, p. 1952) “interpret the ANPS scales as tertiary (thought-mediated) approximations of the influence of the various primary emotional systems in people’s lives.” The same caveats hold for those aiming to study individual differences in raw affect by experimental means. “Pure raw affect” can be overriden by our prefrontally regulated and steered cognitions and behavior action patterns. This makes it very difficult to study individual differences in raw affective responses in humans, but perhaps not impossible. We would like to give some examples for already successful conducted research where more raw forms of affect have been recorded: In adults the study of FEAR had made some progress by using paradigms including the administration of painful electric shocks. One of these paradigms was presented by Mobbs et al. (2007). Here participants were put in a kind of PacMan Game situation, where a predator was chasing the participants. The application of the defensive distance model in this experiment (e.g., Blanchard et al., 2001; McNaughton and Corr, 2004) together with the administration of electric shocks on the hand of the participants, when being caught by the predator, led to a robust switch of more prefrontal monitoring brain activity to “pure” FEAR activity being aroused, as characterized by elevated activity in relevant brain stem regions (e.g., the periaqueductal gray). Other studies used startle reflex paradigms (e.g., Montag et al., 2008) to assess individual differences in the FEAR/anxiety circuitry. Participants differ in their strength of startle reactions to unpredictable administered acoustic loud bursts, a response which obviously cannot be easy controlled by subjects in such experiments. Of course, the study of raw affect in negative primal emotions brings along more ethical issues than the study of positive emotions, which will remain a challenge for university investigators and their ethics committees.

As outlined in this article, we currently have substantial evidence for seven primary emotional systems, which can be evoked by direct electrical stimulation of the brain, as well as various pharmacological challenges to such emotional circuits of mammalian brains. Some of these primal emotions are harder to study than others in humans (just think about social desirability issues when aiming at the study of the raw affect of LUST). Individual differences in other primary emotional systems might require sensitivity of age dependence issues: (i) the separation distress/PANIC system is obviously more active in young children. Such issues are also germane for the study of individual differences in the PLAY circuitry. Young children love to engage in joyful PLAY activity, therefore studying individual differences in PLAY at pure action might be something to be achieved best in the age range of 3–6 (where stable PLAY behavior can be observed, see Scott and Panksepp, 2003). In general, the study of raw affects in children is bound to be easier than in adults, because the prefrontal brain area exerts less influence on the ancient emotional circuitry. Of course, studies of negative emotions in children are confronted by more profound ethical issues than in adults. Of course, the study of raw affect in children is of tremendous importance, because emotional disturbances in childhood have the strongest effect on the development of psychopathological disorders in adults. This is where studies of animal models may be especially informative. Other possibilities (which will give more causal insights into the relevance of primary emotional systems for personality) unfortunately can only be studied in a small group of patients. Anchoring electrodes in areas such as the medial forebrain bundle (the SEEKING system) to treat depression (Coenen et al., 2011; Schlaepfer et al., 2013, 2014; Panksepp et al., 2014) will be also of interest to understand how strong the personality of a patient changes due to the brain stimulation. This approach would also match the investigation of personality changes due to lesions in brain areas where primary emotional systems are concentrated (see example with stroke patients above by Farinelli et al., 2013).

Before concluding this paper we want to mention some limitations of the present theoretical work. First of all, we chose a rather narrow focus in the investigation of primary emotional systems and personality. In the present work we only highlighted the Affective Neuroscience (Panksepp, 1998) perspective on primary emotional systems in the context of the classic Big Five of Personality/Five Factor Model of Personality. Clearly, many other important emotional perspectives could have been chosen such as studying individual differences in emotion recognition and production abilities referring to Ekman’s work on basic emotions derived from faces in the context of personality research. Interestingly this represents also a research area being understudied right now. Please also see how Ekman’s emotion theory (e.g., Ekman and Friesen, 1971) can be linked to Panksepp’s work (Montag and Panksepp, 2016). Aside from Ekman’s theory other emotional theories could have been the focus of the present work such as Russell’s circumplex model (Russell, 1980), which has been contrasted with Panksepp’s work in Zachar and Ellis (2012) or other theorists such as Cal Izard (e.g., Izard, 2009).

A further limitation needs to be named in the context of our evolutionary discussion of personality and their underlying primary emotional systems. E.g., an excellent overview has been provided by Buss (2009) discussing individual differences in personality also in light of the life-history and costly signal theory. Moreover, we could have also addressed the principle of *negative frequency-dependent selection* (the rare phenotype in a population has a selective advantage such as a woman being able to choose among many males in a population with few females), but we refrained from doing this, because “it is in fact just a subcase of the more general phenomenon of fluctuation selection” (Nettle, 2006, p. 625). Of note, personality and intelligence are usually investigated as different phenotypes in psychology. The here-discussed evolutionary *balance selection* criteria might work best for personality and their underlying primary emotional systems, but not for intelligence, where other approaches such as mutation-selection-balance might represent the better approach [Penke et al., 2007; please see for an update the new work by Penke and Jokela (2016)]. For example, perhaps there is simply no disadvantage in being “too” intelligent… but this remains an open issue (e.g., the case of autistic savants).

In closing, we emphasize the importance of building a more solid bridge between human and animal (mammalian) personality research. Such interdisciplinary discourse has diminished markedly since human and animal research traditions parted ways starting half a century ago. In any event, if we want to understand our ancient evolutionary heritage, we have to link individual differences in personality and raw affect that can be studied in animal models as potentially informative of homologies in own brains. We obviously do not have as direct access to such phylogenetically ancient brain systems in humans, except perhaps in neurological studies (Panksepp, 1985). In closing, it will also be worthwhile reconsidering the old works of Darwin on facial expression in men and animals (Darwin, 1872/2008); individual differences in facial emotional expressions, although well studied, could represent a new way to get insights into the dynamics of our human emotional personality dimensions (see discussion in Montag and Panksepp, 2016).

## Ethics Statement

Although this is mainly a theoretical paper, the correlation patterns in the **Tables 1–3** have been derived from three samples consisting of persons filling in questionnaires. As correlation patterns in **Tables 2**, **3** have been not reported before (information from **Table 1** is cited from another study), we note that both for the recruition of participants in Germany and China ethic approval is available.

## Author Contributions

CM drafted the first version of the manuscript. JP worked over this draft, revised it critically and added the section on the relation between emotion and motivation.

## Conflict of Interest Statement

The authors declare that the research was conducted in the absence of any commercial or financial relationships that could be construed as a potential conflict of interest.

## Footnotes

1 Note, that LUST is not assessed with the ANPS, because it is feared that in that kind of scale, answering personal sexuality items may promote the emission of biased social desirable answers, thereby potentially also impacting the psychometric quality of the remaining scales assessing the other primary emotional systems.

2 The Davis et al. (2003) study included Goldberg’s inventory to assess the Big Five, whereas the samples from Germany and China in the present study filled in the NEO-FFI. A study by Mooradian and Nezlek (1996) investigated the correlations between a short version of Goldberg’s inventory and the NEO-FFI concluding: “These results suggest that Saucier’s Mini-Markers and the NEO-FFI measure similar, but clearly not identical constructs. Similarly labeled subscales shared between 25 and 50% of their variance (up to 77% deattenuated)” (Mooradian and Nezlek, 1996, p. 214).

3 In their work they speak of long-billed and deep beaks. We stay with our terminology small/large for reasons of simplicity.
